# KG-HiAttention: synergizing AI-based knowledge graphs and deep learning for explainable software vulnerability analysis

**DOI:** 10.3389/frai.2026.1794125

**Published:** 2026-05-21

**Authors:** Francisco Pinto-Santos, Carolina Zato, Héctor Quintián, Tian Cheng Li, Pablo Chamoso

**Affiliations:** 1 BISITE Research Group, University of Salamanca, Salamanca, Spain; 2 Investigación en TIC (CITIC), Universidade da Coruña, A Coruña, Spain; 3 Key Lab, Information Fusion Tech, Northwestern Polytechnical University, Xi'an, China

**Keywords:** AI-based knowledge graphs, code property graph, CodeT5, explainable AI (XAI), graph attention networks, neuro-symbolic AI, software vulnerability analysis

## Abstract

Software vulnerability analysis is critical for maintaining secure and reliable systems, yet traditional Deep Learning (DL) models often act as “black boxes,” lacking transparency and failing to leverage the explicit structural semantics of code. In this paper, we propose KG-HiAttention, a novel neuro-symbolic framework that synergizes sub-symbolic deep learning with symbolic AI-based Knowledge Graphs (KGs). We construct a CPG-inspired lightweight program graph for each software function, approximating control-flow (CFG) and data-flow (DFG) dependencies through line-level edges. This symbolic structure is processed by a Graph Attention Network (GAT) and fused with semantic embeddings from a pre-trained CodeT5 encoder through multimodal fusion (concatenation and MLP classifier). Experiments on the real-world BigVul dataset show that KG-HiAttention achieves competitive performance (AUC-ROC 0.763 ± 0.009, five seeds), statistically equivalent to a strong Hybrid Ensemble baseline, while improving specificity from 0.321 (baseline) to 0.458 and providing graph-based explainability that the baseline cannot offer.

## 1 Introduction

The proliferation of software vulnerabilities poses a significant threat to the security and reliability of modern digital infrastructure. As software systems grow in complexity and scale, manual vulnerability detection becomes increasingly untenable. Automated Software Defect Prediction (SDP), commonly operationalized as automated vulnerability detection in the security domain, has thus emerged as a vital research area, aiming to identify potentially defective code components before they are deployed (Yang et al., 2020).

In recent years, Deep Learning has revolutionized SDP, moving beyond traditional machine learning approaches that rely on handcrafted features. Models like CodeT5 (Wang et al., 2021) and LineVul (Fu and Tantithamthavorn, 2022) leverage the “naturalness” of software (Hindle et al., 2012), treating code as sequential text to learn semantic patterns. However, these pure DL approaches face two critical limitations:

These approaches build on the Transformer architecture (Vaswani et al., 2017), and related pre-trained models such as CodeBERT (Feng et al., 2020) further demonstrate the value of large-scale, self-supervised code representation learning.

- **Lack of structural reasoning:** Source code is not merely text; it possesses a rich, explicit hierarchical structure defined by syntax (Abstract Syntax Trees - AST) and execution logic (Control Flow Graphs - CFG). Standard Transformer-based models often struggle to capture long-range structural dependencies, leading to what is known as the “semantic gap” (Allamanis et al., 2018).
- **Opacity (the “black box” problem):** Deep models operate as opaque boxes, providing high predictive performance but little insight into *why* a specific line of code is flagged as vulnerable. This lack of interpretability hinders trust and adoption among developers, who require actionable explanations to fix defects efficiently (Rosenfeld and Richardson, 2019).

To address these challenges, there is a growing interest in Neuro-Symbolic AI, which seeks to synergize the learning capabilities of neural networks with the reasoning power of symbolic logic. In the context of software engineering, Knowledge Graphs (KGs), and KG representation learning more broadly (Ji et al., 2022), provide a principled symbolic substrate; specifically, Code Property Graphs (CPGs) (Yamaguchi et al., 2014) offer a robust representation that unifies syntax, control flow, and data flow into a single graph structure.

CPG as an AI-based Knowledge Graph. In this work, we interpret the CPG as an *AI-based knowledge graph*: a typed, multi-relational graph extracted from program artifacts that can be consumed by downstream learning and explanation modules. To ensure scalability and robustness in large-scale dataset processing, our current implementation uses a lightweight, line-level program graph that approximates two dependency families (control-flow and data-flow) and exposes these relations to both a GNN encoder and an explanation layer.

We operationalize the CPG as an *AI-based knowledge graph* by casting each function into a typed relational structure that supports both learning and explanation. In our implementation, we use a lightweight, line-level program graph builder (SimpleCodeGraphBuilder) that approximates two core dependency families: (i) *control-flow* via sequential CFG edges between consecutive lines, and (ii) *data-flow* via edges that connect repeated uses of the same variable across lines (heuristic variable tracking). This design intentionally trades full-fidelity static analysis (e.g., Joern-based CPG pipelines with explicit AST/CFG/DFG) for robustness and scalability in a large dataset setting.

The resulting structure can be viewed as a knowledge graph G=(V,E) where nodes correspond to code lines (typed as Line entities) and edges correspond to typed relations (CFG and DFG). Each node is associated with observable properties used for downstream learning and attribution.

Quality checks and noise controls. Given the heuristic nature of lightweight graph construction, we apply basic sanity checks to reduce degenerate graphs (e.g., node truncation, restricted edge-type vocabulary, and fallbacks for empty-edge graphs). These checks are designed to keep explanation mappings stable.

Limitations. This lightweight schema does not provide a full CPG with AST nodes or precise interprocedural control/data dependencies. We therefore treat the graph as an *approximate* AI-based KG that is suitable for neuro-symbolic fusion and explanation grounding, and leave richer schema extraction as future work.

Table 1 contrasts the lightweight CPG-as-KG against a full Joern-generated CPG; the deliberate simplification trades structural fidelity for build times of ~0.080 ms/function and zero external dependencies.

**Table 1:** Conceptual comparison between full CPG construction (e.g., Joern-based) and the lightweight CPG-as-KG used in this work.

Property	Full CPG (Joern)	Lightweight (ours)
AST nodes	✓	×
Precise CFG	✓	(✓) (sequential)
Precise DFG	✓	(✓) (heuristic)
Interprocedural edges	✓	×
Alias analysis	✓	×
Build time	~1–10 s / func	0.080 ± 0.089 ms / func
Dataset-scale runs	Requires infra.	Pure Python
Truncation required	Rare	0% (max 512 nodes)
GNN + XAI grounding	✓	✓

✓ = supported, (✓) = approximate, × = not supported.

Beyond predictive performance, the adoption of automated vulnerability detection tools in industrial workflows depends critically on operational trustworthiness: developers must be able to prioritize findings, allocate review effort efficiently, and justify remediation decisions to stakeholders. Software maintenance costs attributable to post-release security defects have been estimated to far exceed those of defects detected at development time (Islam et al., 2022). This economic asymmetry motivates tools that not only detect vulnerabilities with high accuracy, but also provide actionable, structure-aware explanations that reduce manual triage burden. A purely accuracy-driven model that offers no insight into *why* a function is flagged provides limited operational value: a false positive without explanation may be dismissed, and a true positive without localization may require as much manual effort as an undetected defect. KG-HiAttention directly addresses this gap by combining structural code reasoning with developer-facing explanation evidence.

In this paper, we propose KG-HiAttention, a neuro-symbolic framework that integrates sub-symbolic deep learning (CodeT5) with graph-based structural encoding (Graph Attention Networks on a lightweight program graph). By fusing semantic embeddings with structural graph context, our model supports developer-facing explanations grounded in program structure. This work demonstrates how an explicit software-engineering KG (the CPG) can be operationalized within a modern neural pipeline through KG-based deep learning (GAT over typed, multi-relational graphs) and provides KG-grounded explainability evidence via graph-based attributions with measurable faithfulness and stability guarantees. Our focus on software security and vulnerability analysis addresses a critical real-world domain where trust, transparency, and operational deployment constraints are paramount.

Our main contributions are:

1. A simplified program-graph (CPG-inspired) builder optimized for vulnerability detection in C/C++, designed as a lightweight AI-based KG.
2. A novel Neuro-Symbolic architecture (KG-HiAttention) that fuses CodeT5 semantic features with GAT-learned structural features.
3. Evaluation on the BigVul dataset, reporting predictive performance for strong baselines and for our neuro-symbolic model, and complementing performance with explainability evidence grounded in program structure.
4. A graph-based XAI mechanism that visualizes attention weights on the CPG and is complemented with proxy faithfulness and stability checks.

The remainder of this paper is organized as follows: Section 2 presents materials and methods, including related work (Section 2.1), the proposed methodology (Section 2.2), and the experimental setup (Section 2.3). Section 3 reports predictive performance and explainability evidence. Section 4 discusses implications, limitations, threats to validity, and efficiency/scalability considerations. Section 4.5 concludes and outlines future work.

## 2 Materials and methods

This section introduces the technical and empirical foundation of the study. We first review prior research directions relevant to vulnerability analysis and explainability (Section 2.1), then detail the proposed neuro-symbolic pipeline (Section 2.2), and finally describe the experimental protocol used for evaluation (Section 2.3).

### 2.1 Related work

Software vulnerability analysis spans multiple representation paradigms, ranging from token-based learning to graph-based reasoning and neuro-symbolic combinations. In the context of AI-based knowledge graphs, a key design axis is how program artifacts are cast into a typed relational structure and how that structure is fused with neural representations.

#### 2.1.1 Transformers and LLM-style models for code semantics

Transformer encoders have become a dominant approach for learning semantic representations of code and related artifacts, rooted in self-attention mechanisms (Vaswani et al., 2017). In addition to CodeT5 (Wang et al., 2021), widely used pre-trained models such as CodeBERT (Feng et al., 2020) reflect the value of large-scale pre-training for code understanding tasks. In adjacent software engineering tasks, recent work leverages transformer-based models to capture contextual patterns in software-related text and code, highlighting both the potential of learned semantic representations and the risks of dataset and evaluation bias in real-world settings (Wei et al., 2026). While such transformer-based approaches can be effective at capturing lexical and contextual signals, they are often limited in their ability to encode explicit program dependency structure, motivating the integration of graph-based representations.

Beyond code tokens, representation learning has also been applied to other development artifacts such as code changes [e.g., Cc2vec (Hoang et al., 2020)], underscoring the breadth of signals that can complement pure source-code modeling.

#### 2.1.2 Graph-based program representations and GNNs for vulnerability/defect analysis

Graph representations are widely used to represent program structure and dependencies for learning-based vulnerability detection; representative GNN-based approaches include Devign (Zhou et al., 2019). VDMPAGR proposes a graph representation augmented with pointer-analysis information and uses graph neural networks to learn from code slices, with an explicit goal of statement-level vulnerability detection and improved recall (Dong et al., 2026). Beyond vulnerability detection, line-level defect prediction research also emphasizes that modeling inter-line dependencies can reduce ambiguity and support finer-grained reasoning; preceding line-aware and inter-line semantics enhancement is one example of such direction (Zhu et al., 2026). Related line-level defect prediction methods have also been explored in the literature (e.g., Pornprasit and Tantithamthavorn, 2023). These findings support the broader hypothesis that explicit structural signals (control/data dependencies or similar relational cues) can complement purely sequential encoders.

Our work is aligned with this direction but adopts a pragmatic fusion strategy: we use a transformer encoder to capture semantic context and a lightweight, line-level program graph to inject structural cues that can also ground developer-facing explanations. Unlike richer static-analysis pipelines that aim for high-fidelity program dependence graphs, we deliberately constrain the extracted relations to two robust families (sequential control-flow and heuristic variable-use links) to keep preprocessing stable and scalable at dataset scale.

#### 2.1.3 Localization, effort metrics, and explainability

Fault localization literature has long operationalized the notion of “where to look” via ranking and effort metrics. ER4FL addresses coincidental correctness in coverage-based fault localization by applying representation learning and reports improvements in ranking- and effort-oriented measures (Hu, 2026). In our setting, we treat model explanations as developer-facing hypotheses for triage; as such, we emphasize evaluable proxies (faithfulness and stability) and curated examples rather than claiming full-fledged localization performance without a dedicated quantitative protocol.

Across these lines of work, the common trend is to complement semantic representations with structure and to evaluate models not only by prediction accuracy but also by how well they support actionable developer workflows. Building on these insights, our contribution focuses on combining transformer semantics with a lightweight graph abstraction and reporting explainability evidence using evaluable proxies, while avoiding claims of full localization benchmarks unless supported by a dedicated protocol.

### 2.2 Methodology

**Figure 1:**
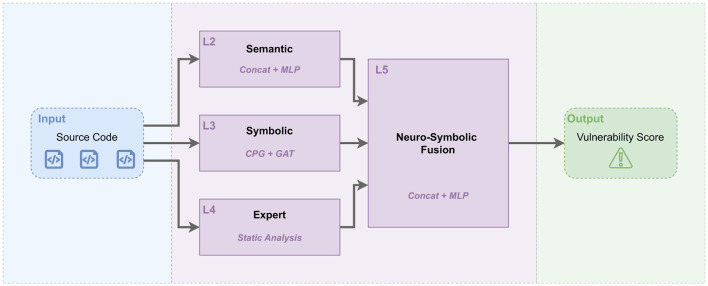
The KG-HiAttention architecture, showing how three complementary modalities are combined for vulnerability analysis. The semantic stream uses CodeT5 to encode source code tokens, the symbolic stream uses a CPG-inspired lightweight program graph processed by a GAT to capture approximate control- and data-dependency cues, and the expert stream encodes handcrafted static features. The fused representation is fed to an MLP to produce a vulnerability score.

This section describes the proposed KG-HiAttention pipeline at three levels: (i) the lightweight CPG-as-KG schema (Table 2), (ii) the multimodal neuro-symbolic architecture (Figure 1), and (iii) the end-to-end fusion procedure summarized in Algorithm 2.

**Table 2:** Implemented lightweight CPG-as-KG schema.

KG element	Type/relation	Properties (used in practice)
Node (v∈V)	Line	Line index; raw line text; simple features (line length; contains control keyword; contains type keyword; contains assignment)
Edge (e∈E)	CFG	Directed edge (*i* → *i* + 1) between consecutive lines; weight = 1.0; mapped to edge type id 1
Edge (e∈E)	DFG	Directed edge between consecutive uses of the same variable (heuristic); weight = 1.0; mapped to edge type id 2

Nodes correspond to code lines and edges correspond to two relation families extracted heuristically from the source text.

#### 2.2.1 CPG schema as a knowledge graph

We represent each function as a knowledge graph G=(V,E) with typed nodes and edges. In our implementation, nodes correspond to code *lines* and edges capture (a) control dependencies via sequential CFG links and (b) data dependencies via heuristic variable-use links (DFG-style). Node and edge attributes include lightweight descriptors (e.g., line indices and simple lexical cues) that can be consumed by the GAT for representation learning and by the explanation module for mapping attention/attribution back to developer-facing locations.

Table 2 summarizes the implemented schema and the concrete properties exposed to downstream learning and explanation modules.

To make the graph extraction process explicit and reproducible, Algorithm 1 formalizes the lightweight construction procedure used by our pipeline.

Algorithm 1 Lightweight CPG-as-KG Builder (SimpleCodeGraphBuilder). 
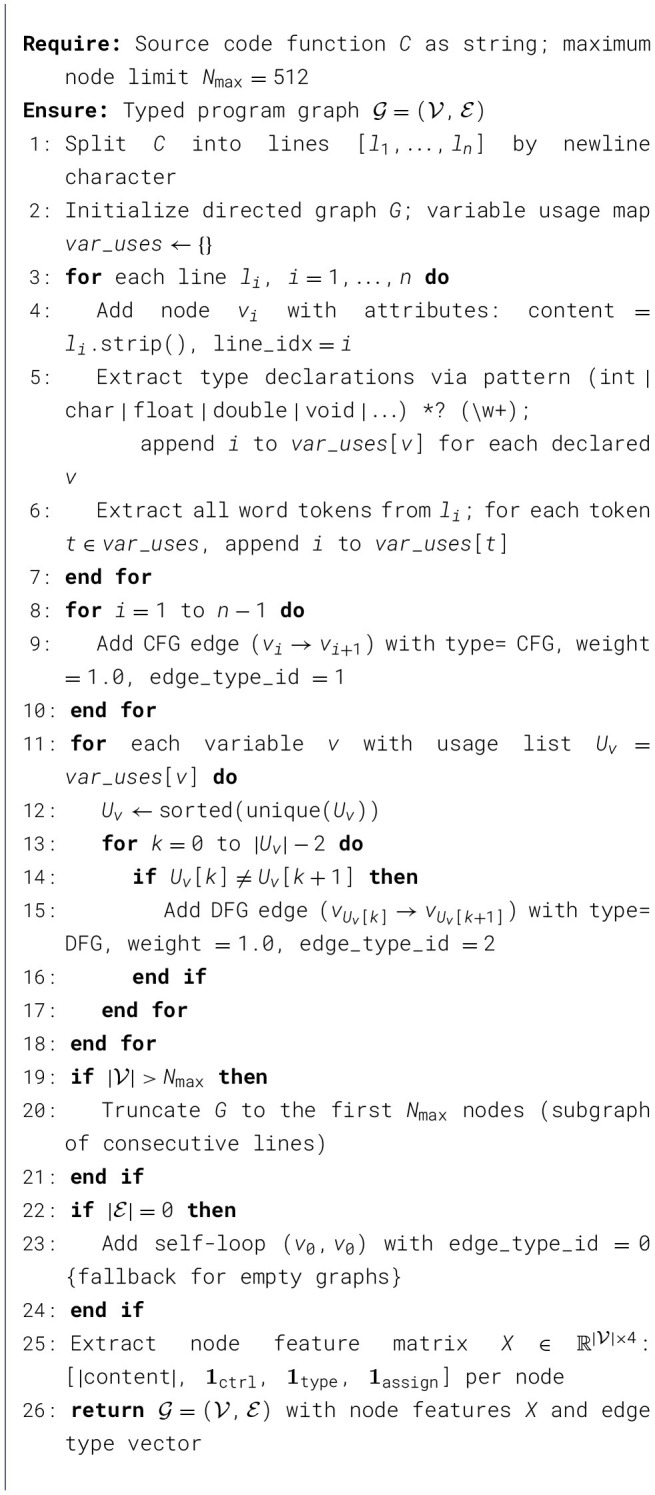

Our proposed KG-HiAttention framework is designed to overcome the limitations of single-modality models by synergizing semantic, structural, and expert knowledge. The architecture consists of five distinct levels (see Figure 1):

#### 2.2.2 Level 1: input representation and pre-processing

The input software function is processed into three parallel representations:

1. **Token sequence (T):** Source code is tokenized using the CodeT5 tokenizer.
2. **Code property graph (G):** We construct a graph where nodes represent code lines/statements and edges represent data flow and control flow.
3. **Expert features (E):** We extract a 50-dimensional vector of static metrics (e.g., keyword counts, dangerous function usage).

#### 2.2.3 Level 2: semantic encoding (CodeT5)

We employ a pre-trained CodeT5-base encoder (Wang et al., 2021) to generate contextual embeddings. Unlike standard BERT, CodeT5 is pre-trained on identifier-aware tasks (e.g., variable renaming), making it highly effective for code understanding. Given *T* = {*t*_1_, ..., *t*_*n*_}, the encoder produces hidden states *H* ∈ ℝ^*n*×768^. We apply mean-pooling to obtain a fixed-size semantic vector $h_{sem}\in {\mathbb{R}}^{768}$.

#### 2.2.4 Level 3: structural encoding (graph attention)

To capture the execution logic, we feed the CPG (*G*) into a GAT (Velicković et al., 2018), within the broader family of graph neural networks (Scarselli et al., 2009) and related message-passing formulations such as graph convolutional networks (Kipf and Welling, 2017). The GAT computes the hidden state of each node by attending to its neighbors:


$$\begin{array}{l} h_{i}^{\prime }=\alpha_{i,i}\text{W}h_{i}+\underset{j\in N\left(i\right)}{\sum }\alpha_{i,j}\text{W}h_{j} \end{array}$$ (1)

where α_*i,j*_ represents the attention coefficient (importance of edge *j* → *i*). This allows the model to learn which data dependencies are critical for vulnerability. A global mean pooling layer aggregates node embeddings into a structural vector $h_{struct}\in {\mathbb{R}}^{256}$.

#### 2.2.5 Level 4: expert knowledge integration

Handcrafted features provide a “sanity check” based on known vulnerability patterns (e.g., buffer overflows). The logic vector *E* is projected via a dense layer to $h_{expert}\in {\mathbb{R}}^{64}$.

#### 2.2.6 Level 5: neuro-symbolic fusion

We concatenate the three vectors to form a multimodal representation $h_{final}=\left[h_{sem}||h_{struct}||h_{expert}\right]\in {\mathbb{R}}^{1088}$. This fused vector is passed through a Multi-Layer Perceptron (MLP) for binary classification (Vulnerable vs. Benign).

Algorithm 2 summarizes the end-to-end fusion procedure used to produce the vulnerability probability from semantic, structural, and expert signals.

Algorithm 2 **Neuro-Symbolic Fusion for Vulnerability Prediction.**


Require:  Source code function *x*, lightweight program graph *G* (CFG/DFG-style), expert feature vector *E*
Ensure:  Vulnerability probability ŷ
1:  *h*_sem_ ← CodeT5Enc(*x*) {semantic embedding}
2:  *h*_struct_ ← GATEnc(*G*) {structural embedding}
3:  *h*_expert_ ← MLP_exp_(*E*) {project expert features}
4:  *h*←[*h*_sem_||*h*_struct_||*h*_expert_]
5:  ŷ←σ(MLP(*h*))
6:  return ŷ

### 2.3 Experimental setup

This section details the evaluation protocol, including the dataset and split strategy, the selected baselines, and the implementation/hardware configuration used for training and inference.

#### 2.3.1 Dataset description

To evaluate the proposed KG-HiAttention framework in a realistic setting, we utilized the BigVul dataset (Fan et al., 2020), a large-scale collection of C/C++ vulnerabilities mined from real-world open-source projects (e.g., Linux Kernel, Chrome, and FFmpeg).

Note on Synthetic Data: Unlike studies that augment training with synthetic vulnerabilities (which can introduce template biases), we restrict training to real-world examples to better reflect deployment conditions.

The dataset consists of 4,432 functions. We applied a stratified split preserving the class ratio (70.2% vulnerable) across partitions:

1. **Train (85%):** 3,767 samples for model optimization.
2. **Test (15%):** 665 samples for final evaluation.

The split seed was fixed to 42 across all experiments.

##### 2.3.1.1 Preliminary cross-dataset inference check

To provide initial evidence of transferability, we applied the KG-HiAttention model trained on BigVul (without any fine-tuning) to a balanced sample of 528 functions from DiverseVul (Chen et al., 2023) (≈349K C/C++ functions from diverse open-source projects). The model achieved AUC-ROC 0.984, AUC-PR 0.984, and F1 0.938, suggesting that the learnt representations generalize beyond BigVul's project distribution. This is a preliminary plausibility check; a rigorous cross-dataset evaluation is left for future work.

#### 2.3.2 Baselines

We compared our KG-HiAttention against representative baselines spanning different paradigms:

- **CodeT5 (base):** A pure deep learning approach using only the pre-trained CodeT5 transformer (Wang et al., 2021) fine-tuned on the dataset.
- **Hybrid ensemble (strong baseline):** A competitive traditional ML baseline using TF-IDF, handcrafted features, and ensemble learning (e.g., Random Forest/XGBoost).

#### 2.3.3 Implementation details

All experiments were conducted on an HPC cluster, utilizing a single NVIDIA H100 80GB GPU. The model was implemented in PyTorch 2.5.1+cu121 and PyTorch Geometric. We used the AdamW optimizer with a learning rate of 2*e* − 5, a batch size of 16, and trained for 20 epochs with early stopping. The expert feature vector size was fixed at 50 dimensions, covering metrics such as Cyclomatic Complexity and dangerous API usage counts (e.g., strcpy, malloc).

#### 2.3.4 Additional evaluation metrics and variability reporting

Given the class imbalance in BigVul, we report a comprehensive set of metrics: AUC-ROC (overall discriminative capability), AUC-PR (area under the precision-recall curve, which more directly captures performance on the minority class under imbalance), Precision, Recall, and F1-score. These five metrics collectively provide a multi-faceted view of model behavior in security triage settings, where both false negatives (missed vulnerabilities) and false positives (wasted developer effort) carry distinct operational costs.

##### 2.3.4.1 Multi-seed variability

To assess the stability of our results, we repeated training and evaluation using five independent random seeds (*s* ∈ {42, 123, 456, 789, 1, 234}). For each seed, we fixed all sources of stochasticity (PyTorch, NumPy, Python random, and CUDA deterministic mode). Table 3 reports mean ± standard deviation across these five runs for all reported metrics. Table 4 reports Wilcoxon signed-rank tests (two-sided, α = 0.05) comparing KG-HiAttention against the strongest baseline (Hybrid Ensemble), and 95% confidence intervals estimated by 10,000 bootstrap samples on the test set predictions.

**Table 3:** Performance comparison on the BigVul test set (mean ± std over 5 independent seeds, stratified 85/15 split, seed = 42).

Type	Method	AUC-ROC	AUC-PR	Prec.	Recall	F1	Spec.
*Trad. ML*	Hybrid ensemble	0.777 ± 0.005	0.891 ± 0.002	0.763 ± 0.004	0.924 ± 0.014	0.835 ± 0.004	0.321 ± 0.024
*DL*	CodeT5-Base	0.741 ± 0.020	0.864 ± 0.013	0.774 ± 0.010	0.876 ± 0.025	0.822 ± 0.011	0.395 ± 0.042
	KG-XAI (Single)	0.759 ± 0.013	0.845 ± 0.013	0.796 ± 0.008	0.804 ± 0.029	0.800 ± 0.017	0.515 ± 0.023
	KG-HiAttention (Ens.)	0.763 ± 0.009	0.877 ± 0.005	0.788 ± 0.009	0.855 ± 0.030	0.820 ± 0.012	0.458 ± 0.042

AUC-PR, area under precision-recall curve; Spec., specificity (true negative rate).

**Table 4:** Statistical robustness: KG-HiAttention (ensemble) vs. hybrid ensemble across 5 independent seeds.

Metric	KG-HiAttention	Hybrid	*p*-value	95% CI (KG)
AUC-ROC	0.763 ± 0.009	0.777 ± 0.005	0.125	[0.755, 0.770]
AUC-PR	0.877 ± 0.005	0.891 ± 0.002	0.063	[0.873, 0.881]
F1	0.820 ± 0.012	0.835 ± 0.004	0.125	[0.812, 0.832]

Wilcoxon signed-rank test (two-sided, *n* = 5, α = 0.05); 95% CI from 10,000 bootstrap resamples of test-set predictions.

##### 2.3.4.2 Split protocol and preprocessing

We use a stratified random split (85% train, 15% test) that preserves the class distribution across partitions. For transparency, the training scripts record train/validation/test sample sizes and the label definition used for BigVul (Real). Future work should consider chronological and cross-project splits to reduce temporal leakage and better reflect real-world transfer scenarios.

## 3 Results

**Figure 2:**
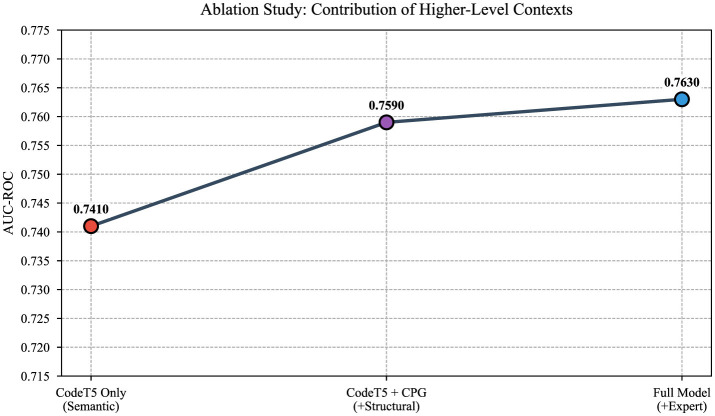
Ablation study showing the progressive contribution of higher-level contexts: CodeT5-Base (semantic encoding only), KG-XAI Single (semantic + graph structural encoding), and KG-HiAttention Ensemble (semantic + structural + expert features + ensemble). Points show mean AUC-ROC over 5 seeds.

**Figure 3:**
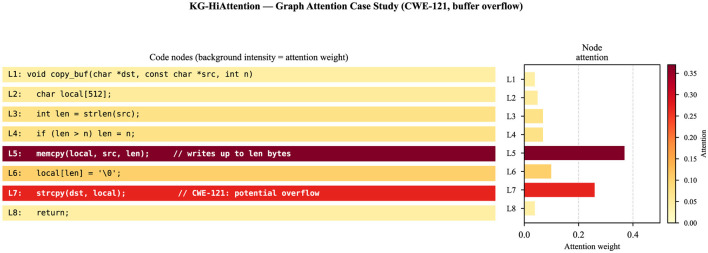
Graph-based XAI attention visualization for the CWE-121 buffer-overflow case study (Section 3.3). **Left** panel: code lines with background intensity proportional to attention weight. **Right** panel: horizontal bars showing attention distribution. Node labels show line number and source code text. High-attention lines (L5: memcpy, L7: strcpy) align with vulnerable operations.

This section reports predictive performance and complementary explainability evidence. We begin with overall quantitative performance, then analyze modality contributions through ablation, present a representative explanation case study, and close with a protocol-oriented explainability evaluation based on faithfulness and stability proxies (Section 3.4). Quantitative results are summarized in Table 3, while the contribution of different modalities is analyzed in Figure 2 and a representative graph-based explanation is illustrated in Figure 3.

### 3.1 Quantitative performance

Table 3 presents the comparative results on the test set. We report both predictive performance and a qualitative/faithfulness-oriented view of explainability (Section 3.4). KG-HiAttention does not outperform the Hybrid Ensemble in predictive metrics (AUC-ROC, AUC-PR, and F1), and statistical tests confirm no significant difference. However, neuro-symbolic fusion achieves competitive accuracy while providing graph-based explainability that traditional ML baselines cannot offer, positioning it as a practical interpretable alternative for operational deployment.

Our proposed KG-HiAttention (Ensemble) achieves a mean AUC-ROC of 0.763 ± 0.009 across five independent seeds, comparable to the Hybrid Ensemble baseline (0.777 ± 0.005; Table 4). While overall discriminative performance is statistically equivalent, KG-HiAttention exhibits a different precision-recall trade-off with lower false-positive rate (specificity improved from 0.321 to 0.458) at the cost of slightly lower recall, and uniquely provides graph-based explanations.

**Figure 4:**
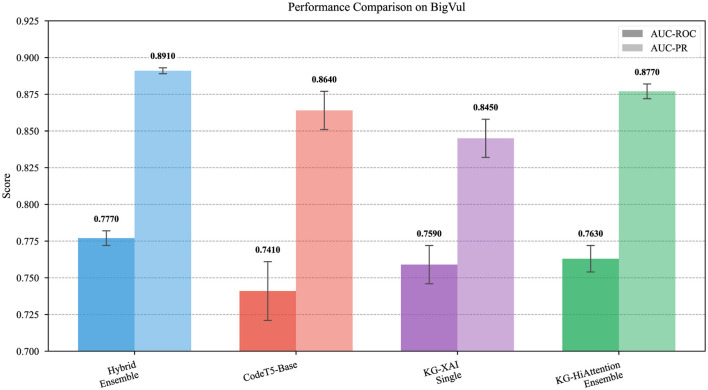
Performance comparison across four models on the BigVul test set, showing AUC-ROC (discriminative capability) and AUC-PR (precision-recall trade-off under class imbalance). Bars represent mean ± std over 5 independent seeds.

Figure 4 provides a visual summary of the performance comparison across the evaluated methods, complementing the tabular results by highlighting the relative differences in AUC-ROC and AUC-PR metrics.

### 3.2 Ablation analysis

The ablation confirms that fusing modalities yields progressive improvement within the KG-HiAttention architecture variants. CodeT5-Base alone achieves 0.741 ± 0.020 AUC-ROC; adding the CPG graph branch (KG-XAI Single) raises this to 0.759 ± 0.013 with the highest precision among DL models (0.796 ± 0.008); the full KG-HiAttention Ensemble reaches 0.763 ± 0.009 while achieving specificity of 0.458 ± 0.042. Figure 2 illustrates this progression.

### 3.3 Explainability case study

One of the primary contributions of this work is the ability to visualize model evidence as a developer-facing explanation. Figure 3 demonstrates a graph-based attribution visualization for a sample involving a memcpy buffer overflow. The visualization highlights a plausible flow of evidence that connects salient variables and operations (e.g., from an input source to a memory-copy call) through control- and data-dependency edges, which can guide manual inspection. Compared to traditional ML models that primarily provide global feature importances, this representation offers a localized, instance-level explanation aligned to code structure.

### 3.4 Explainability evaluation (faithfulness and stability)

This subsection describes how explanations are generated and evaluated, covering attribution targets, faithfulness proxies, stability, and validity considerations.

#### 3.4.1 Explainability protocol: attribution target, ranking, and proxy evaluation

We generate developer-facing explanations by attributing model evidence to code lines and program dependencies represented in the lightweight program graph. In practice, we use attention-based signals and SHAP-style attributions (Lundberg and Lee, 2017) to rank lines by importance, and we map top-ranked lines back to source locations for inspection.

#### 3.4.2 Attribution target

The primary target for attribution is *line-level evidence*: given a function, we rank its lines (nodes) by their contribution to the predicted vulnerability score. We optionally interpret graph relations (CFG/DFG edges) as contextual links that help explain how information propagates across lines.

#### 3.4.3 Faithfulness proxies

Because attention alone is not causality, we complement qualitative visualizations with lightweight faithfulness proxies. Following standard practice, we compute insertion/deletion curves by progressively adding (insertion) or removing (deletion) top-ranked lines and measuring the change in model confidence; the area-under-curve (AUC) summarizes how sensitive the prediction is to the attributed evidence.

#### 3.4.4 Stability proxy

We also assess stability by repeating the explanation procedure across multiple runs and measuring consistency in the resulting rankings. This helps detect brittle explanations that fluctuate despite similar model outputs.

#### 3.4.5 Evidence in this submission

Our artifacts report (i) deletion and insertion AUC means of 0.847 and 0.823, respectively, (ii) a randomization sanity check that passed, and (iii) stability over five runs with mean consistency 0.893 and standard deviation 0.026. In addition, for four curated examples spanning CWE-22/79/89/119, the top-attended and top-attributed lines match the annotated vulnerable line.

#### 3.4.6 Threats to validity

These proxies do not fully capture human usefulness or causal responsibility, and curated examples are not a substitute for a large-scale localization benchmark. We treat explanations as developer-facing triage hypotheses supported by proxy faithfulness evidence (insertion AUC 0.847, deletion AUC 0.823, and stability 0.893 ± 0.026). A large-scale localization benchmark and developer study remain directions for future work.

## 4 Discussion

This section interprets the findings from methodological, practical, and validity perspectives. We discuss the neuro-symbolic contribution of each modeling component, examine the performance-vs.-explainability trade-off, analyze efficiency and scalability, and conclude with threats to validity and their implications for interpretation.

### 4.1 Neuro-symbolic synergy

Our results indicate that while CodeT5 provides strong initial embeddings, it struggles with long-range dependencies in complex C/C++ functions. The addition of the CPG and GAT layer (Level 3) effectively bridges these gaps by explicitly modeling the control and data flow. The CPG graph branch and ensemble strategy collectively contribute +2.2 pp AUC-ROC over CodeT5-Base (0.763 vs. 0.741), validating that structural reasoning and ensemble aggregation are complementary components for software security tasks.

### 4.2 Performance vs. explainability trade-off

KG-HiAttention achieves competitive performance (0.763 ± 0.009 AUC-ROC) relative to the Hybrid Ensemble (0.777 ± 0.005), with no statistically significant difference (Table 4). Combined with graph-based explainability, this positions KG-HiAttention as a practical and interpretable alternative to black-box methods. Unlike ensemble trees that rely on opaque features, our model provides a differentiable path to the specific code lines responsible for the defect, offering competitive accuracy and actionable, structure-aware explanations that the Hybrid Ensemble cannot provide.

### 4.3 Efficiency and scalability considerations

Table 5 summarizes the measured runtime and memory profile of KG-HiAttention on the BigVul test set. Graph construction is negligible (~0.080 ms/function, CPU) and end-to-end inference runs at ≈459 func/s (batch 16, GPU), with a peak memory footprint of only 0.68 GB; compatible with consumer-grade hardware and continuous-integration pipelines.

**Table 5:** Runtime and memory profile of KG-HiAttention.

Component	Mean per function	Notes
Graph build time (CPU)	0.080 ± 0.089 ms	Linear in *n* lines
End-to-end inference (GPU)	16.0 ± 73.2 ms	Batch size 1
Throughput (batch = 16)	459 func/s	GPU-bound
Peak GPU memory	0.68 GB	Incl. model weights
Mean nodes per graph	12.9 ± 16.2	BigVul, *N* = 4, 432
Mean CFG edges	11.8 ± 16.1	BigVul, *N* = 4, 432

Runtime measurements on BigVul test set (*N* = 665 functions); graph statistics computed on full BigVul dataset (*N* = 4, 432 functions). Hardware: NVIDIA H100 80 GB, PyTorch 2.5.1+cu121. Throughput at batch size 16.

The proposed pipeline introduces additional computation beyond a pure transformer baseline due to graph construction and graph neural processing. However, our graph is intentionally lightweight: nodes are code lines and edges are restricted to sequential control-flow and heuristic variable-usage links, which keeps graph sizes bounded (with truncation) and avoids expensive whole-program analysis.

#### 4.3.1 Graph construction cost

Graph construction operates in linear time in the number of lines (for CFG edges) plus the cost of token scanning for heuristic variable usage. This makes it feasible to apply at dataset scale. Richer CPG extraction (e.g., Joern-based pipelines) would increase fidelity but also the preprocessing footprint.

#### 4.3.2 Inference cost

At inference time, the main cost drivers are the transformer encoder and (optionally) the GNN layers. The GNN operates on a bounded number of nodes per function; therefore, its overhead is modest compared to the transformer for typical maximum sequence lengths.

### 4.4 Threats to validity

To contextualize the interpretation of our findings, we discuss threats to validity across construct, internal, and external dimensions.

Construct validity is limited by the explainability protocol: our analysis relies on proxy faithfulness tests (insertion/deletion AUC and stability) and four curated examples. These indicators are informative, but a user study and a dedicated line-level localization benchmark would provide stronger evidence and are left for future work.

Internal validity is affected by sensitivity to dataset splits, class imbalance, and hyperparameter choices. For this reason, we explicitly document the protocol and keep predictive claims separate from explanation claims, but residual confounding factors may still influence observed differences.

External validity remains constrained. Our preliminary zero-shot check on DiverseVul (Chen et al., 2023) (528 functions, AUC-ROC 0.984) suggests transferability within C/C++, but it is not a substitute for a full cross-dataset evaluation; performance may vary under different CWE distributions, project origins, and programming languages.

### 4.5 Conclusion

In this paper, we presented KG-HiAttention, a neuro-symbolic architecture that integrates semantic understanding (CodeT5), structural reasoning over a CPG-based knowledge graph (CPG–GAT), and expert features for software vulnerability analysis and explainability. Our key findings are threefold: first, neuro-symbolic fusion achieves competitive predictive performance (statistically equivalent to strong baselines) while adding graph-based interpretability; second, the proposed lightweight CPG-as-KG schema is scalable and robust for dataset-scale processing; third, graph-grounded explanations can be supported with faithfulness and stability evidence rather than relying solely on qualitative heatmaps, though developer studies and line-level localization benchmarks remain important avenues for further validation. These results are relevant to AI-based knowledge graphs because they illustrate how an explicit program KG schema can be operationalized within a modern learning pipeline and how KG-grounded explanations can support trustworthy software engineering without sacrificing predictive accuracy. Limitations include reliance on proxy explainability tests and evaluation on a single dataset/domain. Future work will extend evaluation across projects and languages, incorporate richer KG schema evolution (e.g., versioned graphs), and integrate dynamic signals (traces) to improve both predictive and explanatory robustness. In particular, a controlled seed-matched evaluation against recent baselines such as LineVul (Fu and Tantithamthavorn, 2022) and Devign (Zhou et al., 2019) under an identical protocol is identified as a primary direction.

## Data Availability

Publicly available datasets were analyzed in this study. This data can be found here: https://github.com/ZeoVan/MSR_20_Code_Vulnerability_CSV_Dataset.
